# 1-Carb­oxy­naphthalen-2-yl acetate monohydrate

**DOI:** 10.1107/S1600536813034338

**Published:** 2014-01-04

**Authors:** Bruno S. Souza, Adailton J. Bortoluzzi, Faruk Nome

**Affiliations:** a Depto. de Química – Universidade Federal de Santa Catarina, 88040-900 Florianópolis, Santa Catarina, Brazil

## Abstract

In the title compound, C_13_H_10_O_4_·H_2_O, both the carboxylic acid [C_ar_—C_ar_—C—O = −121.1 (2)°, where ar = aromatic] and the ester [C_ar_—C_ar_—O—C = −104.4 (3)°] groups lie out of the mean plane of the conjugated aromatic system. In the crystal, the organic mol­ecule is hydrogen bonded to water mol­ecules through the ester and carb­oxy moieties, forming chains along the *a*-axis direction. The methyl H atoms of the acet­oxy group are disordered over two equally occupied sites.

## Related literature   

For the synthesis, see: Chattaway (1931 ▶). For related structures, see: Souza *et al.* (2007 ▶, 2010 ▶); Fitzgerald & Gerkin (1993 ▶). For effects of the spatial relationship between reacting groups on the mechanism and speed of intra­molecular reactions, see: Orth *et al.* (2010 ▶). For hydrolysis mechanisms, see: Souza & Nome (2010 ▶). 
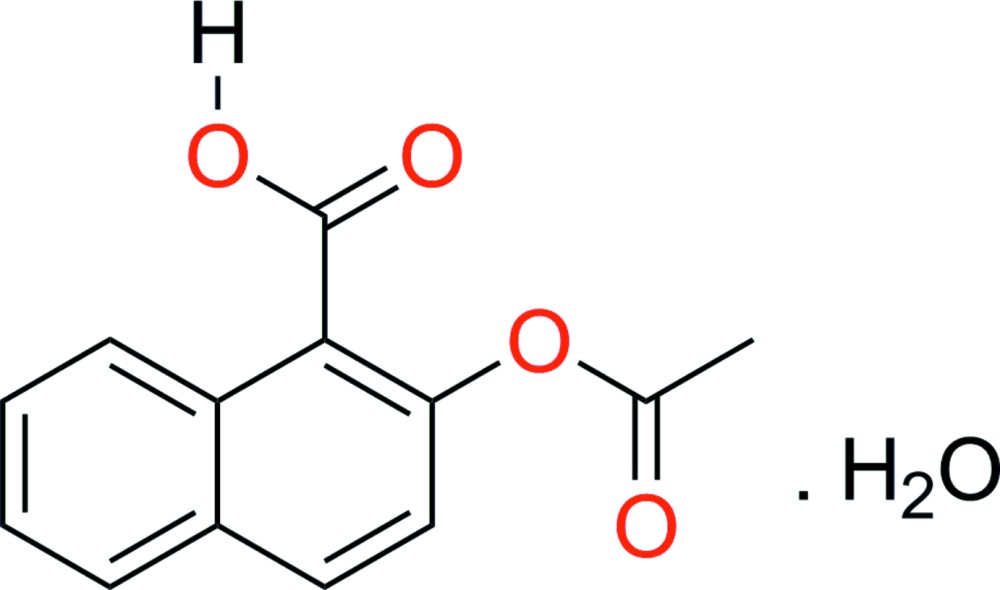


## Experimental   

### 

#### Crystal data  

C_13_H_10_O_4_·H_2_O
*M* *_r_* = 248.23
Monoclinic, 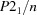

*a* = 9.0539 (4) Å
*b* = 11.6668 (6) Å
*c* = 11.8297 (19) Å
β = 94.863 (10)°
*V* = 1245.1 (2) Å^3^
*Z* = 4
Mo *K*α radiation
μ = 0.10 mm^−1^
*T* = 293 K
0.43 × 0.33 × 0.26 mm

#### Data collection  

Enraf–Nonius CAD-4 diffractometer
2405 measured reflections
2294 independent reflections
1298 reflections with *I* > 2σ(*I*)
*R*_int_ = 0.021
3 standard reflections every 200 reflections intensity decay: 1%

#### Refinement  

*R*[*F* ^2^ > 2σ(*F* ^2^)] = 0.045
*wR*(*F* ^2^) = 0.125
*S* = 1.05
2294 reflections
175 parameters
H atoms treated by a mixture of independent and constrained refinement
Δρ_max_ = 0.15 e Å^−3^
Δρ_min_ = −0.12 e Å^−3^

### 

Data collection: *CAD-4 Software* (Enraf–Nonius, 1989 ▶); cell refinement: *SET4* in *CAD-4 Software*; data reduction: *HELENA* (Spek, 1996 ▶); program(s) used to solve structure: *SIR97* (Altomare *et al.*, 1999 ▶); program(s) used to refine structure: *SHELXL2013* (Sheldrick, 2008 ▶); molecular graphics: *PLATON* (Spek, 2009) ▶; software used to prepare material for publication: *SHELXL2013*.

## Supplementary Material

Crystal structure: contains datablock(s) global, I. DOI: 10.1107/S1600536813034338/hg5366sup1.cif

Structure factors: contains datablock(s) I. DOI: 10.1107/S1600536813034338/hg5366Isup2.hkl

Supporting information file. DOI: 10.1107/S1600536813034338/hg5366Isup3.cdx

Supporting information file. DOI: 10.1107/S1600536813034338/hg5366Isup4.cml

CCDC reference:

Additional supporting information:  crystallographic information; 3D view; checkCIF report

## Figures and Tables

**Table 1:** Hydrogen-bond geometry (Å, °)

*D*—H⋯*A*	*D*—H	H⋯*A*	*D*⋯*A*	*D*—H⋯*A*
O3—H3⋯O1*W*	0.96 (4)	1.64 (4)	2.585 (3)	167 (3)
O1*W*—H1*WA*⋯O2^i^	0.91 (4)	1.81 (4)	2.697 (3)	165 (3)
O1*W*—H1*WB*⋯O4^ii^	0.87 (4)	1.93 (4)	2.754 (3)	158 (3)

Symmetry codes: (i) 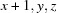; (ii) 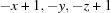.

**Fig. 1.**
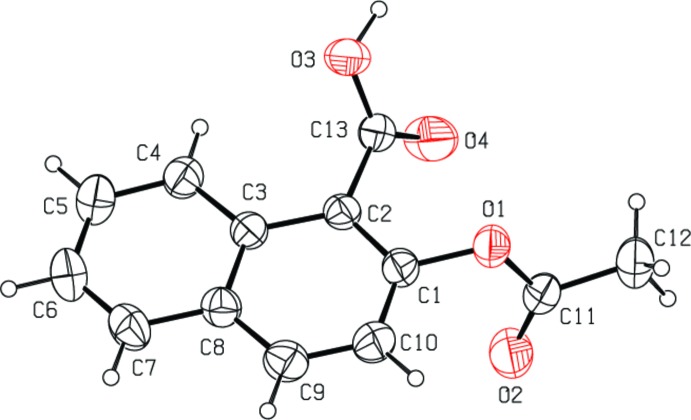
The molecular structure of the title compound with labeling scheme. Displacement ellipsoids are shown at the 40% probability level.

**Fig. 2.**
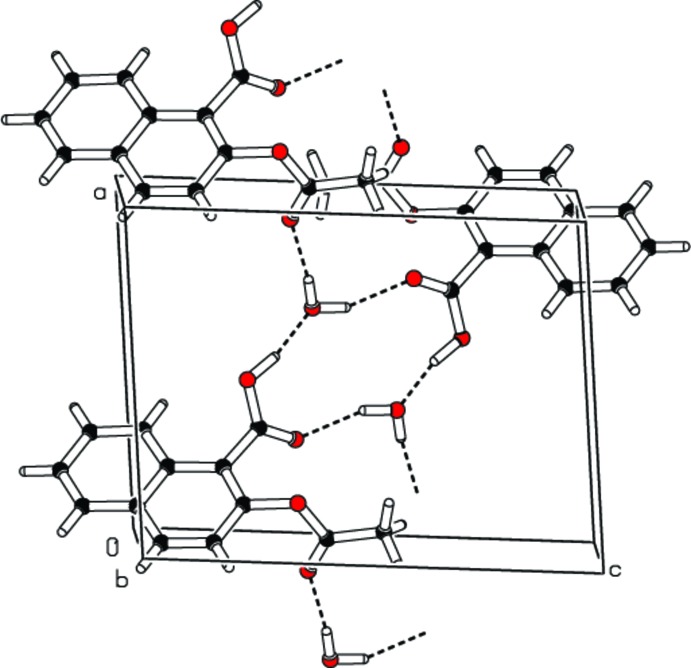
One-dimensional polymer parallel to a axis formed by hydrogen bonds.

## supplementary crystallographic information

### 1. Comment

It has been extensively shown that the spatial relationship between reacting groups have drastic effects on the mechanism and speed of intramolecular reactions (Orth *et al.*, 2010). Recently, we have reported the structure of 2-carboxy-1-naphtyl acetate (Souza *et al.*, 2007) and 3-acetoxy-2-naphthoic acid (Souza *et al.*, 2010), constitutional isomers of the title compound. In a detailed experimental and theoretical investigation, it has been shown that although 2-carboxy-1-naphthyl acetate and 3-acetoxy-2-naphthoic acid show similar structures, they display very different hydrolysis mechanisms (Souza & Nome, 2010). In the current report, we show the crystal structure of 1-carboxy-2-naphthyl acetate (I) which may be a useful molecule for further investigations related to proximity and orientation effects.

A projection of the crystal structure of (I) is shown in Fig. 1. The carboxy group lies out of the mean aromatic plane, with a C1—C2—C13—O3 torsion angle of -121.1 (2)°, while in the structure of 1-naphthoic acid the equivalent torsion is 7.73° (Fitzgerald & Gerkin, 1993). Similarly, the acetyl group lies almost perpendicular to the aromatic ring, with C2—C1—O1—C11 tosrion angle of -104.4 (3)°. The organic fragment is hydrogen bonded to water molecules with interactions centered in both the COOH group and the acetyl group forming one-dimensional polymeric structure parallel to crystallographic *a* axis (Fig. 2).

### 2. Experimental

The title compound was prepared by following the procedure reported in the literature (Chattaway, 1931). In an Erlenmeyr flask containing a magnetic bar, 100 ml of water, 1.40 g of KOH and 2.24 g of 2-hydroxy-1-naphthoic acid were dissolved. The liquid was cooled and mixed with crushed ice. Under vigorous mixing, 1.40 ml of acetic anhydride was quickly added, forming a white precipitate. The reaction was allowed to warm to room temperature, acidified with aqueous HCl, and the white material was filtered. After recrystallization in aqueous ethanol a white powder that melts at 375–376 K was obtained. Crystals suitable for X-ray diffraction were obtained by dissolving about 10 mg of the as prepared material in 5 ml of CHCl_3_ in a 10 ml glass vial and the flask was kept in a saturated petroleum ether atmosphere at 293 K.

### 3. Refinement

All non-H atoms were refined with anisotropic displacement parameters. H atoms were placed at their idealized positions with distances of 0.93 Å for C—H_Ar_ and 0.96 Å for CH_3_ group. Their *U*_eq_ were fixed at 1.2 and 1.5 times *U*_iso_ of the preceding atom for aromatic and methyl group, respectively. H atoms of the methyl group were added as idealized disordered over two positions. The hydrogen atoms of the acid group and water molecule were located from the Fourier difference map and treated as free atoms.

### Crystal data

**Table:**

C_13_H_10_O_4_·H_2_O	*F*(000) = 520
*M**_r_* = 248.23	*D*_x_ = 1.324 Mg m^−^^3^
Monoclinic, *P*2_1_/*n*	Mo *K*α radiation, λ = 0.71073 Å
*a* = 9.0539 (4) Å	Cell parameters from 25 reflections
*b* = 11.6668 (6) Å	θ = 6.9–15.5°
*c* = 11.8297 (19) Å	µ = 0.10 mm^−^^1^
β = 94.863 (10)°	*T* = 293 K
*V* = 1245.1 (2) Å^3^	Irregular block, colorless
*Z* = 4	0.43 × 0.33 × 0.26 mm

### Data collection

**Table:**

Enraf–Nonius CAD-4 diffractometer	θ_max_ = 25.5°, θ_min_ = 2.5°
Radiation source: fine-focus sealed tube	*h* = −10→10
ω–2θ scans	*k* = −14→0
2405 measured reflections	*l* = −14→0
2294 independent reflections	3 standard reflections every 200 reflections
1298 reflections with *I* > 2σ(*I*)	intensity decay: 1%
*R*_int_ = 0.021	

### Refinement

**Table:**

Refinement on *F*^2^	0 restraints
Least-squares matrix: full	Hydrogen site location: mixed
*R*[*F*^2^ > 2σ(*F*^2^)] = 0.045	H atoms treated by a mixture of independent and constrained refinement
*wR*(*F*^2^) = 0.125	*w* = 1/[σ^2^(*F*_o_^2^) + (0.0419*P*)^2^ + 0.2858*P*] where *P* = (*F*_o_^2^ + 2*F*_c_^2^)/3
*S* = 1.05	(Δ/σ)_max_ < 0.001
2294 reflections	Δρ_max_ = 0.15 e Å^−^^3^
175 parameters	Δρ_min_ = −0.12 e Å^−^^3^

### Fractional atomic coordinates and isotropic or equivalent isotropic displacement parameters (Å^2^)

**Table:**

	*x*	*y*	*z*	*U*_iso_*/*U*_eq_	Occ. (<1)
C1	0.0923 (3)	0.2036 (2)	0.23934 (19)	0.0595 (6)	
C2	0.1906 (2)	0.12872 (19)	0.19971 (18)	0.0539 (6)	
C3	0.1849 (2)	0.1063 (2)	0.08057 (18)	0.0551 (6)	
C4	0.2802 (3)	0.0283 (2)	0.0315 (2)	0.0692 (7)	
H4	0.3520	−0.0105	0.0775	0.083*	
C5	0.2677 (3)	0.0094 (3)	−0.0827 (2)	0.0823 (8)	
H5	0.3319	−0.0418	−0.1136	0.099*	
C6	0.1608 (4)	0.0655 (3)	−0.1536 (2)	0.0860 (9)	
H6	0.1539	0.0518	−0.2313	0.103*	
C7	0.0673 (3)	0.1397 (3)	−0.1098 (2)	0.0798 (8)	
H7	−0.0043	0.1765	−0.1578	0.096*	
C8	0.0757 (3)	0.1627 (2)	0.0079 (2)	0.0623 (7)	
C9	−0.0210 (3)	0.2410 (2)	0.0553 (2)	0.0756 (8)	
H9	−0.0915	0.2794	0.0076	0.091*	
C10	−0.0136 (3)	0.2617 (2)	0.1685 (2)	0.0725 (8)	
H10	−0.0779	0.3136	0.1983	0.087*	
C11	0.0021 (3)	0.1791 (2)	0.4176 (2)	0.0693 (7)	
C12	0.0281 (3)	0.2029 (3)	0.5404 (2)	0.0893 (9)	
H12A	0.1143	0.2504	0.5541	0.134*	0.5
H12B	0.0433	0.1319	0.5809	0.134*	0.5
H12C	−0.0564	0.2416	0.5660	0.134*	0.5
H12D	−0.0468	0.1656	0.5799	0.134*	0.5
H12E	0.0241	0.2840	0.5531	0.134*	0.5
H12F	0.1239	0.1743	0.5680	0.134*	0.5
C13	0.3018 (3)	0.0725 (2)	0.2822 (2)	0.0618 (7)	
O1	0.10370 (18)	0.22872 (15)	0.35630 (13)	0.0694 (5)	
O2	−0.0957 (2)	0.1226 (2)	0.37330 (17)	0.1085 (8)	
O1W	0.6442 (2)	0.0245 (2)	0.4136 (2)	0.0825 (6)	
O3	0.4388 (2)	0.09187 (19)	0.26311 (16)	0.0817 (6)	
O4	0.2668 (2)	0.0182 (2)	0.36196 (17)	0.1059 (8)	
H1WA	0.727 (4)	0.068 (3)	0.407 (3)	0.118 (12)*	
H1WB	0.647 (4)	0.012 (3)	0.486 (3)	0.127 (14)*	
H3	0.504 (4)	0.063 (3)	0.325 (3)	0.141 (14)*	

### Atomic displacement parameters (Å^2^)

**Table:**

	*U*^11^	*U*^22^	*U*^33^	*U*^12^	*U*^13^	*U*^23^
C1	0.0575 (15)	0.0678 (17)	0.0537 (14)	−0.0080 (13)	0.0062 (12)	−0.0035 (12)
C2	0.0487 (13)	0.0602 (15)	0.0528 (14)	−0.0071 (12)	0.0043 (11)	0.0028 (11)
C3	0.0535 (14)	0.0590 (15)	0.0530 (14)	−0.0050 (12)	0.0053 (11)	0.0028 (11)
C4	0.0688 (17)	0.0746 (18)	0.0646 (17)	0.0084 (14)	0.0089 (13)	0.0004 (13)
C5	0.098 (2)	0.084 (2)	0.0668 (19)	0.0066 (17)	0.0193 (16)	−0.0065 (16)
C6	0.112 (3)	0.096 (2)	0.0515 (16)	−0.001 (2)	0.0101 (17)	−0.0051 (16)
C7	0.091 (2)	0.091 (2)	0.0554 (16)	−0.0025 (17)	−0.0053 (14)	0.0094 (15)
C8	0.0632 (16)	0.0653 (17)	0.0583 (15)	−0.0013 (13)	0.0042 (12)	0.0086 (12)
C9	0.0721 (18)	0.081 (2)	0.0727 (18)	0.0140 (15)	−0.0005 (14)	0.0116 (15)
C10	0.0676 (18)	0.0748 (19)	0.0755 (19)	0.0118 (14)	0.0079 (14)	−0.0024 (14)
C11	0.0619 (16)	0.085 (2)	0.0627 (16)	−0.0018 (15)	0.0152 (14)	−0.0094 (14)
C12	0.100 (2)	0.106 (2)	0.0635 (17)	−0.0008 (19)	0.0171 (16)	−0.0147 (16)
C13	0.0554 (16)	0.0776 (18)	0.0527 (14)	0.0008 (13)	0.0065 (11)	0.0023 (13)
O1	0.0672 (11)	0.0819 (12)	0.0603 (11)	−0.0111 (9)	0.0124 (9)	−0.0138 (9)
O2	0.0853 (14)	0.162 (2)	0.0806 (14)	−0.0544 (15)	0.0216 (11)	−0.0253 (14)
O1W	0.0611 (13)	0.1075 (16)	0.0773 (14)	−0.0148 (12)	−0.0026 (10)	0.0156 (12)
O3	0.0539 (11)	0.1188 (17)	0.0716 (12)	−0.0007 (11)	−0.0001 (9)	0.0153 (11)
O4	0.0798 (14)	0.153 (2)	0.0846 (15)	−0.0001 (14)	0.0071 (11)	0.0543 (14)

### Geometric parameters (Å, º)

**Table:**

C1—C2	1.359 (3)	C9—H9	0.9300
C1—C10	1.394 (3)	C10—H10	0.9300
C1—O1	1.409 (3)	C11—O2	1.190 (3)
C2—C3	1.430 (3)	C11—O1	1.349 (3)
C2—C13	1.493 (3)	C11—C12	1.478 (3)
C3—C4	1.412 (3)	C12—H12A	0.9600
C3—C8	1.416 (3)	C12—H12B	0.9600
C4—C5	1.364 (4)	C12—H12C	0.9600
C4—H4	0.9300	C12—H12D	0.9600
C5—C6	1.390 (4)	C12—H12E	0.9600
C5—H5	0.9300	C12—H12F	0.9600
C6—C7	1.345 (4)	C13—O4	1.201 (3)
C6—H6	0.9300	C13—O3	1.299 (3)
C7—C8	1.414 (3)	O1W—H1WA	0.91 (4)
C7—H7	0.9300	O1W—H1WB	0.87 (4)
C8—C9	1.413 (4)	O3—H3	0.96 (4)
C9—C10	1.357 (4)		
			
C2—C1—C10	122.9 (2)	O2—C11—O1	121.1 (2)
C2—C1—O1	118.5 (2)	O2—C11—C12	126.0 (3)
C10—C1—O1	118.6 (2)	O1—C11—C12	112.9 (2)
C1—C2—C3	119.2 (2)	C11—C12—H12A	109.5
C1—C2—C13	118.8 (2)	C11—C12—H12B	109.5
C3—C2—C13	122.0 (2)	H12A—C12—H12B	109.5
C4—C3—C8	118.0 (2)	C11—C12—H12C	109.5
C4—C3—C2	123.4 (2)	H12A—C12—H12C	109.5
C8—C3—C2	118.6 (2)	H12B—C12—H12C	109.5
C5—C4—C3	120.6 (3)	C11—C12—H12D	109.5
C5—C4—H4	119.7	H12A—C12—H12D	141.1
C3—C4—H4	119.7	H12B—C12—H12D	56.3
C4—C5—C6	121.1 (3)	H12C—C12—H12D	56.3
C4—C5—H5	119.4	C11—C12—H12E	109.5
C6—C5—H5	119.4	H12A—C12—H12E	56.3
C7—C6—C5	119.9 (3)	H12B—C12—H12E	141.1
C7—C6—H6	120.0	H12C—C12—H12E	56.3
C5—C6—H6	120.0	H12D—C12—H12E	109.5
C6—C7—C8	121.3 (3)	C11—C12—H12F	109.5
C6—C7—H7	119.4	H12A—C12—H12F	56.3
C8—C7—H7	119.4	H12B—C12—H12F	56.3
C9—C8—C7	122.0 (2)	H12C—C12—H12F	141.1
C9—C8—C3	119.0 (2)	H12D—C12—H12F	109.5
C7—C8—C3	119.1 (2)	H12E—C12—H12F	109.5
C10—C9—C8	121.7 (2)	O4—C13—O3	123.2 (2)
C10—C9—H9	119.1	O4—C13—C2	122.5 (2)
C8—C9—H9	119.1	O3—C13—C2	114.2 (2)
C9—C10—C1	118.7 (2)	C11—O1—C1	116.24 (19)
C9—C10—H10	120.6	H1WA—O1W—H1WB	103 (3)
C1—C10—H10	120.6	C13—O3—H3	110 (2)
			
C10—C1—C2—C3	−1.1 (3)	C2—C3—C8—C9	1.5 (3)
O1—C1—C2—C3	−177.20 (19)	C4—C3—C8—C7	−0.5 (3)
C10—C1—C2—C13	178.8 (2)	C2—C3—C8—C7	−178.9 (2)
O1—C1—C2—C13	2.7 (3)	C7—C8—C9—C10	179.1 (3)
C1—C2—C3—C4	−178.7 (2)	C3—C8—C9—C10	−1.3 (4)
C13—C2—C3—C4	1.4 (3)	C8—C9—C10—C1	−0.1 (4)
C1—C2—C3—C8	−0.4 (3)	C2—C1—C10—C9	1.3 (4)
C13—C2—C3—C8	179.7 (2)	O1—C1—C10—C9	177.5 (2)
C8—C3—C4—C5	0.7 (4)	C1—C2—C13—O4	55.8 (4)
C2—C3—C4—C5	179.1 (2)	C3—C2—C13—O4	−124.3 (3)
C3—C4—C5—C6	−0.5 (4)	C1—C2—C13—O3	−121.1 (2)
C4—C5—C6—C7	−0.1 (5)	C3—C2—C13—O3	58.8 (3)
C5—C6—C7—C8	0.4 (5)	O2—C11—O1—C1	−3.9 (4)
C6—C7—C8—C9	179.5 (3)	C12—C11—O1—C1	175.8 (2)
C6—C7—C8—C3	−0.1 (4)	C2—C1—O1—C11	−104.4 (3)
C4—C3—C8—C9	179.9 (2)	C10—C1—O1—C11	79.3 (3)

### Hydrogen-bond geometry (Å, º)

**Table:**

*D*—H···*A*	*D*—H	H···*A*	*D*···*A*	*D*—H···*A*
O3—H3···O1*W*	0.96 (4)	1.64 (4)	2.585 (3)	167 (3)
O1*W*—H1*WA*···O2^i^	0.91 (4)	1.81 (4)	2.697 (3)	165 (3)
O1*W*—H1*WB*···O4^ii^	0.87 (4)	1.93 (4)	2.754 (3)	158 (3)

Symmetry codes: (i) *x*+1, *y*, *z*; (ii) −*x*+1, −*y*, −*z*+1.
